# Further Report of the Committee of Inquiry

**Published:** 1916-12-09

**Authors:** 


### NATIONAL INSURANCE ACT DEVELOPMENTS.

### Further Report of the Committee of Inquiry.

IN THE HOSPITAL for November 18, page 141, we drew attention to what seemed to us to be the unbusinesslike delay in the publication of the Report of the Departmental Committee of Inquiry on approved society finance and administration. While we still hold that the delay has resulted in the creation of undesirable opposition, which threatens to jeopardise the whole scheme of reform, and which might have been avoided if more business-like promptitude had been exercised, we are bound to recognise and admit, now that the further report has been published, that the Committee have accomplished the task of investigation with consummate skill, and that the recommendations as to the simplification of administration are framed on lines which will command general, if not unqualified, approval.

The report is a lengthy document, extending to 459 numbered paragraphs, the last 60 of which are devoted to a summary of the findings and recommendations. In these circumstances we cannot attempt to condense the report within the confines of a single article, but must adopt the plan of selecting for comment such portions as appear to be of outstanding interest to our readers.

### INSURED PERSONS WHO ARE INMATES OF HOSPITALS.

The first of such questions to which we naturally turn is that which refers to the payment of sickness or disablement benefit to, or in respect of, an insured person who is an inmate of a hospital or similar institution. The provisions relating to such circumstances are contained at present under section 12 of the Act of 1911 and section 15 of the Act of 1913, and the Committee recommend that they should be amended in such a manner as to provide "that, so long as an insured person is resident in an institution, no payment of sickness, maternity, or disablement benefit shall be made except under the following conditions:—

(i) Where he has dependants it may be paid to them, or otherwise applied for their benefit, by his society, if he so desires.

(ii) Where he has no dependants, it may, on his authorisation and at the discretion of the society—

(*a*) be utilised, in whole or in part, to defray any expenses, otherwise than in respect of treatment and maintenance, for which he may be, or become, liable while he is in the institution; or, so far as not so utilised,

(*b*) be paid over to the authorities of the institution (not being a Poor-Law institution or other institution maintained out of public funds) under an agreement entered into by his society either generally or in the particular case."

It will at once be apparent that the words here employed have been very carefully chosen, and it would not be surprising if they were adopted as they stand as the text of the clause in the Amendment Bill, when this is presented to Parliament. The words undoubtedly bear a strong resemblance to those used in the sections of the existing Acts, and it is necessary to examine them with some scrutiny to discover the points of importance wherein they differ. We are assisted in this by the statements made by the Committee leading up to their recommendation.

### THE GENERAL OBJECT OF THE PROVISIONS.

The general object of the special provisions in the existing Acts was to adjust the interests of the insured person who is an inmate of an institution, and those dependent upon him, on the one hand, with the interests of the institution on the other hand, subject to the important qualification that State Insurance moneys must not go in relief of the Poor Law. Under the existing law, the insured person and (or) his dependants is given a first charge on the benefit. Subject to this charge, provision is made under which the benefit might, if the hospital authorities and the society agree, and in the absence of dependants, be paid over to the hospital towards the maintenance of the insured person therein. The comment of the Committee on the latter portion of the provision is that they understand that it has not been widely used.

They then go on to illustrate various difficulties that have arisen in the interpretation of the sections. It is not always easy, they say, to determine whether a particular institution is one to which the sections apply; while the question of "dependency" is one which requires particularly careful handling, and as investigations have to be made in each individual case, friction and delay are frequently inevitable. These are difficulties, however, which, in the opinion of the Committee, can be overcome by suitable administrative arrangements.

### FUNDAMENTAL CHANGES REQUIRED.

There are other difficulties inherent in the existing law which cannot be overcome except by a fundamental change. The first of these results from the provision that the benefit *shall* be applied for the relief of dependants, if the insured person has any, or *may* be paid to the authorities of the institution, subject to certain safeguards, if he has no dependants. In no circumstances can the money be applied for his own personal benefit while he remains an inmate of the institution. This is rightly characterised as a serious defect, for thereby many an insured person has been precluded from obtaining necessary surgical appliances until after his discharge from the institution, while others have suffered hardship in not being able to defray personal expenses, such as house rent. The remedy proposed is incorporated in the recommendation already quoted, in which it will be observed that provision is specifically made that "when an insured person has no dependants, the benefit may be utilised to defray expenses . . . for which he may be, or may become, liable while he is in the institution."

After-Care of Persons Leaving Sanatoria.

Under the provisions, as they at present stand, it is necessary when an insured person who has no dependants is an inmate of a sanatorium for the benefit to be paid to the Insurance Committee. Sanatoria are differentiated in this respect from other institutions referred to in the section. The result is that on discharge from a sanatorium the insured person frequently finds himself without adequate means for his own support just at a time when he is in [illegible] special care and nourishment. The Committee therefore recommend that this difference between sanatoria and other institutions should be abrogated, and, consequently, their recommendation as to the application of benefit moneys omits any special reference to the case of insured persons without dependants, who are inmates of sanatoria.

General Repeal not Recommended.

The Committee indicate in their report that they had under consideration whether the difficulties would not be met by repealing the whole of the existing legislation on this part of the subject, and leaving the approved societies a free hand to apply the benefits as thought most desirable in each individual case. They considered, however, that if left entirely to their own discretion approved societies would frequently be subjected to undesirable pressure to pay the benefits in ways not best suited to the interests of the members. A large number of resolutions were submitted by Poor-Law Authorities requesting that an alteration should be made in the practice of accumulating the benefit accruing while the insured person is an inmate of such an institution, and paying it in a lump sum on his discharge. While agreeing that the payment of such lump sums is liable to abuse, as affording the means of self-indulgence to be followed by a return to the institution, the Committee are unable to agree in any way with the possibility that National Insurance moneys should be applied in the relief of local rates. They hold that the abuse should be controlled by the application of the rules of the approved societies, and thus they recommend that the existing provisions of the Acts for the payment to an insured person of any unexpended balance of accumulated benefit moneys, upon his discharge from an institution, should remain unaltered.

Agreements between Hospitals and Approved Societies.

There can be little doubt that, as the result of this careful review, the Committee have produced a recommendation that is a vast improvement upon the existing arrangements. One further point seems to call for attention. It is still only possible for benefit moneys to be paid over to hospital authorities provided an agreement has been entered into between the authority concerned and the particular approved society of which the inmate is a member. Moreover, behind this provision there is the further double qualification that the payment must be authorised by the inmate, and be subject to the discretion of his society. It does not, therefore, follow, even when a general agreement has been entered into between a particular society and a hospital for the treatment of members, that the benefit moneys will be paid over. General agreements would, therefore, seem to be of little or no value, and it is not surprising, in the circumstances, that, as we have already pointed out, the Committee should state that they find this provision has been little used. There is, however, some ground for hope that more use will be made of the provision, for it is now specified that the agreement may be either general, or made in the particular case. This has, from a very early stage, been the interpretation placed upon the existing section by the Insurance Commissioners; but there is reason to suppose that approved societies and hospital authorities have alike been under the impression that general agreements have been requisite, and this may in some measure account for the small number of agreements that have been entered into. We fear, however, that the principal difficulty rests upon the interpretation of the word "dependants," and so long as it is possible for there to be *ad hoc* "dependants," hospital authorities cannot expect to receive any considerable sum of money from National Insurance sources for the extremely valuable services they render in the treatment of insured persons.

